# Impact of dynamic changes of elevated bilirubin on survival in patients on veno-arterial extracorporeal life support for acute circulatory failure

**DOI:** 10.1371/journal.pone.0184995

**Published:** 2017-10-19

**Authors:** Miriam Freundt, Dirk Lunz, Alois Philipp, Bernd Panholzer, Matthias Lubnow, Christine Friedrich, Leopold Rupprecht, Stephan Hirt, Assad Haneya

**Affiliations:** 1 Dept. of Cardiothoracic Surgery, University Medical Center of Regensburg, Regensburg, Germany; 2 Dept. of Anesthesiology and Critical Care, University Medical Center of Regensburg, Regensburg, Germany; 3 Dept. of Cardiovascular Surgery, University of Schleswig-Holstein, Campus Kiel, Kiel, Germany; 4 Dept. of Internal Medicine II, University Medical Center of Regensburg, Regensburg, Germany; Medizinische Hochschule Hannover, GERMANY

## Abstract

**Aims:**

Veno-arterial extracorporeal life support (ECLS) is an established method to stabilize acute circulatory failure. Parameters and data on when to ideally wean circulatory support are limited. Bilirubin is a marker of end-organ damage. Therefore, the purpose of this large study was to evaluate the impact of dynamic changes of elevated bilirubin levels on survival in patients on ECLS.

**Methods and results:**

We reviewed 502 consecutive cases of ECLS from 2007 to 2015. Bilirubin levels were recorded before implantation and until six days after explantation. Dynamic bilirubin changes, and hemodynamic and laboratory outcome parameters were compared in survivors and nonsurvivors. Reason for ECLS implantation was cardiac arrest with ongoing resuscitation in 230 (45.8%), low cardiac output in 174 (34.7%) and inability to wean off cardiopulmonary bypass in 98 (19.5%) patients. 307 (61.2%) patients were weaned off ECLS, however, 206 (41.0%) survived. Mean duration of ECLS was 3 (2–6) days, and survivors received significantly longer ECLS (5 vs 3 days, p < 0.001). Survivors had significantly lower baseline bilirubin levels (*p = 0*.*003*). Bilirubin started to rise from day 2 in all patients. In survivors, bilirubin levels had trended down on the day of ECLS explantation and stayed at an acceptable level. However, in weaned patients who did not survive and patients who died on ECLS bilirubin levels continued to rise during the recorded period.

**Conclusion:**

ECLS support improves survival in patients with acute circulatory failure. Down trending bilirubin levels on veno-arterial ECLS indicate improved chances of successful weaning and survival in hemodynamically stable patients.

## Introduction

Veno- arterial extracorporeal life support (ECLS) is a widely used and well-established method to stabilize patients with acute circulatory failure [1]. It provides gas exchange across a membrane and blood circulation through a pump resulting in immediate organ perfusion upon initiation. ECLS catheters can be placed with ultrasound-guidance and Seldinger technique [2]. In life threatening situations the underlying pathology may not immediately be apparent. Stabilization of circulation can allow the clinician to establish a diagnosis and treatment plan. ECLS can be used as emergency rescue therapy or as bridge to diagnosis and decision. It can serve as temporary support system to await organ recovery or to bridge until a more permanent device or transplantation becomes available [1,3–5]. By providing immediate tissue perfusion ECLS offers a chance of recovery from end organ damage. Long-term survival with acceptable quality of life has been reported [6].

Several studies evaluated initial risk factors for mortality on ECLS [6–10]. But, limited parameters exist to guide weaning from ECLS or to indicate readiness for explantation. One recent Japanese trial confirmed that weaning from ECLS does not necessarily result in survival [11]. This trial did not evaluate parameters for weaning. In clinical practice we had observed improved outcome in patients with relatively lower bilirubin levels. Thus, the aim of this study was to evaluate the prognostic value of dynamic changes in bilirubin levels on survival while on ECLS for acute circulatory failure. To our knowledge, this is the first study to investigate this in a large population of over 500 patients on ECLS.

## Material and methods

### Study design

We performed a retrospective analysis of 502 cases from the University Medical Center Regensburg ECLS registry. The registry is maintained prospectively and anonymized for internal quality assurance. Cases are included with all-comers design. Individual patients were not consented and approval by the institutional review board was waived due to the retrospective design with data from an anonymized internal registry. This is consistent with our institutional review boards requirements. We reviewed all data, and the information we have not shared is actually not needed to interpret this study.

### Study population

From 2007 until November 2015, 502 consecutive adult patients underwent ECLS for acute circulatory failure, including cardiac arrest, low cardiac output state (LCO) and inability to wean from extracorporeal circulation (ECC) after cardiac surgery. In all of those scenarios the indication for initiation of ECLS per our institutional protocol is hemodynamic instability with evidence of insufficient organ perfusion despite inotropic and vasopressor support. We had no exclusion criteria for case review.

Patients’ baseline characteristics, laboratory data, clinical variables, hemodynamic profiles and outcome data were gathered from the institutional quality assurance ECLS registry. Hemodynamic parameters and change of laboratory variables were recorded before ECLS implantation and daily until six days after explantation.

Primary endpoints were successful weaning from ECLS and survival. Survival was defined as discharge from the hospital. Secondary endpoints were complications, switch to left ventricular assist device (LVAD) or veno-venous extracorporeal membrane oxygenation (VV ECMO), heart transplantation, blood transfusion and cause of death. Successful weaning was defined as explantation of ECLS with subsequent hemodynamic stability. Non-survival was defined as death from any cause while on ECLS or during hospitalization after successful weaning. We routinely consider ECLS if no return of spontaneous circulation occurs within 15 minutes of conventional cardiopulmonary resuscitation (CPR) [5]. Initiation of ECLS can be achieved at bedside or in the emergency department via percutaneous ultrasound-guided cannulation. We commonly accessed femoral artery and vein, subclavian and internal jugular veins were cannulated rarely. If peripheral percutaneous access was not possible, surgical central cannulation via the ascending aorta and right atrium was performed. All cases transitioned from ECC to ECLS were cannulated centrally.

Our institution serves as a statewide referral center for ECLS. Patients were transferred from surrounding community hospitals if eligible for ECLS. In case of impending circulatory failure our team travelled to the referring hospital or road accident site for initiation of portable ECLS [4].

### ECLS systems

During the study period four different ECLS systems were used: Permanent Life Support (PLS) System and Cardiohelp (MAQUET Cardiopulmonary AG, Rastatt, Germany), Hilite7000LT oxygenator + DP3 pump (Medos Medizintechnik, Stolberg, Germany), and ECC.05 system (Sorin Group, Modena, Italy). All four systems are portable with a priming volume of less than 600 cc’s normal saline. Details about cannulas, management and anticoagulation are described in our previous paper [12].

### Patient management

Initial ECLS pump flow was set to maintain stable mean arterial pressure of 55–65 mmHg. The fraction of inspired oxygen was set to 100% and the ECLS sweep gas flow was adjusted based on carbon dioxide levels in post-oxygenator blood gas analysis. Indicator of sufficient tissue perfusion was a venous blood gas saturation (drawn from a port before the membrane oxygenator of >70%). Diagnostic and therapeutic interventions including radiographic (computed tomography) or angiographic (coronary angiography) imaging were performed on ECLS as soon as possible.

After treatment of the underlying disease and stabilization of hemodynamics ECLS was weaned. Extracorporeal blood flow was gradually reduced to 1.5L/min depending on hemodynamic stability, need for vasopressor support and laboratory markers. If all remained stable, percutaneous cannulas were manually removed at bedside and pressure was applied. In obese patients or patients with difficult implantation or severe arteriosclerosis the arterial cannula was removed surgically. Surgically implanted central cannulas were removed in the operating room.

### Statistical analysis

**Fig 1:**
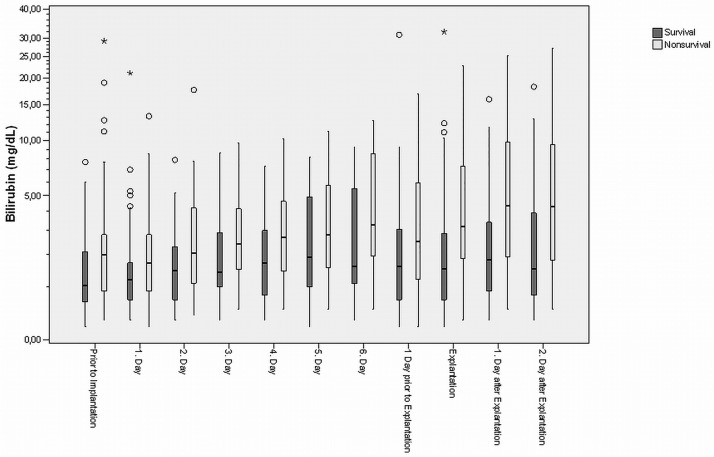
The boxplots show the correlation between bilirubin levels and time course in survivors and non-survivors. (+ = significant after Bonferroni-Holm correction; ★ = extreme values [> 3-fold box length]; ○ = outlier [> 1.5-fold box length])

**Fig 2:**
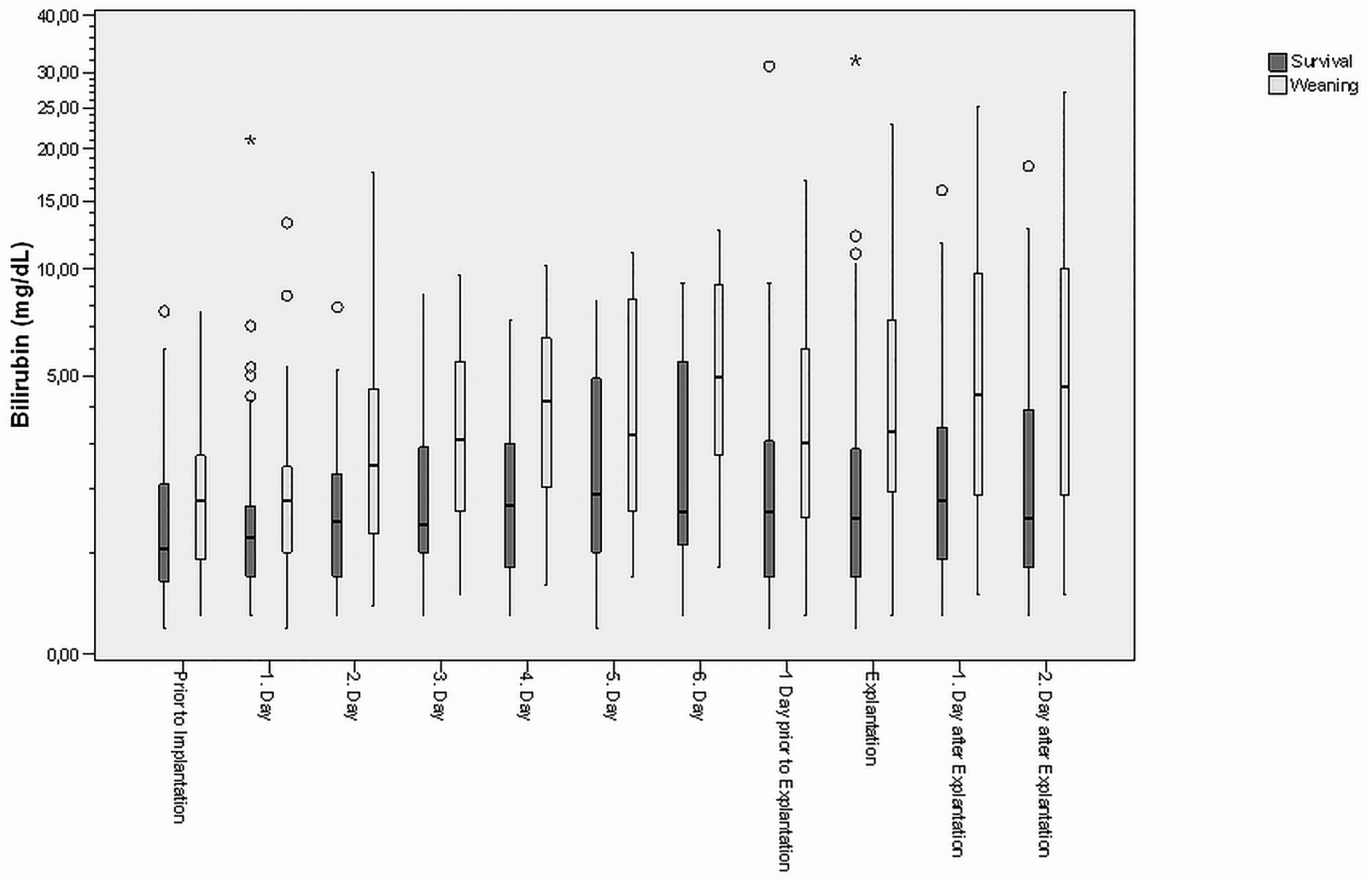
The boxplots show the correlation between bilirubin levels and time course in weaned patients who did not survive. (+ = significant after Bonferroni-Holm correction; ★ = extreme values [> 3-fold box length]; ○ = outlier [> 1.5-fold box length])

SPSS 16.0 (SPSS, Chicago, IL) and Stata SE 10.1 (StatCorp, College Station, TX) were use. Patient characteristics are described by descriptive statistics. Means and standard deviations were computed for normally distributed continuous variables. Student *t* test was applied to compare normally distributed data. Non-normally distributed continuous data were described by means and interquartile ranges (25^th^– 75^th^) and compared by Man-Whitney *U* test. Categorical variables are shown as frequency distributions (*n*) and percentages *(%)*. Chi-square test and Fisher exact test were performed for univariate comparison between groups with categorical variables. A multivariable regression analysis was performed to determine independent risk factors for mortality. Boxplots were generated for bilirubin levels. The Bonferroni-Holm correction was used to reduce the chance of type I error in multiple testing (Figs 1 and 2). The probability of survival was determined on the basis of survival curves using the Kaplan–Meier method and compared using the log-rank test. Statistical significance was determined as *p* values < 0.05.

## Results

Baseline characteristics are listed in Table 1. Statistically significant differences between groups were: a higher Sequential Organ Failure Assessment (SOFA) score in non-survivors (11 vs 13, *p < 0*.*001*), a lower body mass index in survivors (26 vs 27, *p = 0*.*004*). Coronary artery disease was more frequent in non-survivors [144 (48.6%) vs 64 (31.1%); *p < 0*.*001*], cardiomyopathy [88 (42.7%) vs 86 (29.1%); *p = 0*.*002*], and trauma were more frequent in survivors [8 (3.9%) vs 2 (0.07%); *p = 0*.*02*]. Cardiac surgery prior to ECLS was more frequently seen in non-survivors [126 (42.6% vs 68 (33.0%); *p = 0*.*03*].

**Table 1:** Baseline characteristics of the study population.

	All patients	Survival	Non-survival	*p* Value
No. of patients, n (%)	502 (100%)	206 (41.0%)	296 (59%)	
Age, years	59 ± 15 (61; 50–70)	58 ± 13 (59; 50–67)	59 ± 15 (62; 51–71)	0.08
Gender male, n (%)	369 (73.5%)	150 (72.8%)	219 (74.0%)	0.77
Body mass index, kg/m^2^	27 (24–30)	26 (24–29)	27 (25–31)	*0*.*004*
SOFA score	12 (10–14)	11 (9–14)	13 (11–15)	*<0*.*001*
Lung injury score	2.7 (2.0–3.0)	2.5 (1.7–3.0)	2.7 (2.0–3.0)	0.216
Underlying disease, n (%)				
Cardiac	424 (84.5%)	165 (80.1%)	259 (87.5%)	*0*.*03*
Coronary artery disease Valve disease Cardiomyopathy	208 (41.4%) 42 (8.4%) 174 (34.7%)	64 (31.1%) 13 (6.3%) 88 (42.7%)	144 (48.6%) 29 (9.8%) 86 (29.1%)	*<0*.*001* 0.19 *0*.*002*
Non-cardiac	78 (15.5%)	41 (19.9%)	37 (12.5%)	*0*.*03*
Pulmonary embolism ARDS Accidental hypothermia Trauma Others	26 (5.2%) 18 (3.5%) 5 (1.0%) 10 (2.0%) 19 (3.8%)	13 (6.3%) 10 (4.8%) 2 (1.0%) 8 (3.9%) 8 (3.9%)	13 (4.4%) 8 (2.7%) 3 (1.0%) 2 (0.07%) 11 (3.7%)	0.41 0.23 1.0 *0*.*02* 0.98
Cardiac surgery prior to ECLS, n (%)	194 (38.6%)	68 (33.0%)	126 (42.6%)	*0*.*03*
Previous CPR, n (%)	369 (73.5%)	146 (70.9%)	223 (75.3%)	0.30
RRT prior to ECLS, n (%)	114 (22.7%)	39 (18.9%)	75 (25.3%)	0.10

Table 2 outlines ECLS implantation data. 230 (45.8%) patients received ECLS implantation for cardiac arrest with ongoing CPR. [survivors, n = 85 (41.3%) vs. non-survivors, n = 145 (48.9%); *p = 0*.*1*]. Approximately one third (n = 174, 34.7%) of the patients received ECLS for LCO, with a significantly higher survival [88 (42.7%) vs 86 (29.1%); *p = 0*.*002*]. 98 (19.5%) required ECLS for ongoing support after cardiac surgery, with significantly less survival [33 (16.0%) vs 65 (22.0%); *p = 0*.*004*]. 117 (23.3%) ECLS systems were implanted outside our center, with an increased survival [67 (32.5%) vs 50 (16.9%); *p < 0*.*001*]. There was no significant difference with regard to peripheral cannulation sites. Non-survivors were more frequently cannulated centrally via right atrium [40 (13.5%) vs 13 (6.3%); *p = 0*.*01*] and ascending aorta [63 (21.3%) vs 28 (13.6%); *p = 0*.*03*].

**Table 2:** ECLS implantation data of the study population.

	All patients (n = 502)	Survival (n = 206)	Non-survival (n = 296)	*p* Value
Reason for ECLS implantation, n (%)				
CPR LCO No weaning from ECC	230 (45.8%) 174 (34.7%) 98 (19.5%)	85 (41.3%) 88 (42.7%) 33 (16.0%)	145 (48.9%) 86 (29.1%) 65 (22.0%)	0.10 *0*.*002* *0*.*004*
Out-of-center implantation, n (%)	117 (23.3%)	67 (32.5%)	50 (16.9%)	*<0*.*001*
Cannulation site venous, n(%)				
Femoral vein Right atrium Jugular vein Subclavian artery	428 (85.3%) 53 (10.6%) 13 (2.6%) 8 (1.6%)	181 (87.9%) 13 (6.3%) 7 (3.4%) 5 (2.4%)	247 (83.4%) 40 (13.5%) 6 (2.0%) 3 (1.0%)	0.20 *0*.*01* 0.40 0.28
Cannulation site arterial, n (%)				
Femoral artery Subclavian artery Ascending aorta	372 (74.1%) 39 (7.8%) 91 (18.1%)	159 (77.2%) 19 (9.2%) 28 (13.6%)	213 (72.0%) 20 (6.8%) 63 (21.3%)	0.21 0.31 *0*.*03*
Percutaneous cannulation, n (%)	361 (71.9%)	150 (72.8%)	211 (71.3%)	0.76
Surgical cannulation, n (%)	141 (28.1%)	56 (27.2%)	85 (28.7%)	0.76

Table 3 lists the hemodynamic parameters and change of laboratory variables before and one day after ECLS implantation. Most hemodynamic parameters had improved on day 1 with reduction in norepinephrine and epinephrine requirements. Survivors had significantly higher mean arterial pressure initially and on day one (*p = 0*.*03 and p < 0*.*001*), lower doses of norepinephrine (*p = 0*.*008*) and epinephrine *(p = 0*.*002*) drips on day one, and lower lactate levels initially and on day one (*p < 0*.*001*). Bilirubin levels were lower initially and on day one in survivors (*p = 0*.*003* and *p = 0*.*013*). Platelets were higher in survivors initially and on day one (*p < 0*.*001* and *p = 0*.*005*).

**Table 3:** Hemodynamic parameters and change of laboratory variables before and one day after ECLS implantation.

	All patients (n = 502)	Survival (n = 206)	Nonsurvival (n = 296)	*p* Value
Mean arterial pressure, mmHg				
T0 T1	55 (45–67) 66 (61–73)	57 (45–68) 68 (62–75)	54 (40–65) 65 (59–72)	0.03 *<0*.*001*
Norepinephrine, mg/h				
T0 T1	1.8 (1.0–3.0) 0.4 (0.0–1.0)	2.0 (1.0–3.5) 0.3 (0.0–0.9)	1.8 (1.0–3.0) 0.5 (0.1–1.0)	0.171 *0*.*008*
Epinephrine, mg/h				
T0 T1	1.0 (0.1–2.0) 0.3 (0.1–0.7)	0.9 (0.0–2.0) 0.3 (0.1–0.5)	1.0 (0.3–2.0) 0.4 (0.2–0.9)	0.191 *0*.*002*
apH				
T0 T1	7.25 (7.13–7.35) 7.42 (7.35–7.48)	7.25 (7.17–7.36) 7.44 (7.39–7.49)	7.25 (7.08–7.33) 7.39 (7.33–7.47)	0.075 *<0*.*001*
Lactate, mg/dl				
T0 T1	76 (44–119) 42 (21–80)	62 (39–98) 27 (17–52)	87 (54–142) 68 (30–126)	*<0*.*001* *<0*.*001*
Bilirubin, mg/dl				
T0 T1	1.6 (0.7–2.6) 1.3 (0.7–2.5)	1.1 (0.6–2.1) 1.2 (0.7–1.7)	2.0 (0.9–2.9) 1.7 (0.9–2.9)	*0*.*003* *0*.*013*
GOT, U/l				
T0 T1	170 (56–401) 489 (185–1245)	133 (44–380) 359 (123–883)	183 (63–435) 573 (232–1558)	0.058 *<0*.*001*
LDH, U/l				
T0 T1	465 (274–797) 872 (476–1660)	417 (248–675) 736 (420–1261)	533 (298–841) 971 (568–2234)	*0*.*008* *<0*.*001*
Hemoglobin, g/dl				
T0 T1	10.2 (8.6–12.0) 9.6 (9.0–10.6)	10.4 (8.8–12.2) 9.5 (8.9–10.6)	9.9 (8.4–11.9) 9.6 (9.0–10.6)	0.069 0.637
Plateltes, /nl				
T0 T1	178 (124–243) 119 (82–169)	198 (138–257) 129 (91–173)	161 (113–233) 107 (69–156)	*<0*.*001* *0*.*005*

Outcome parameters are shown in Table 4. Mean duration of ECLS therapy was 3 (2–6) days, and survivors received significantly longer ECLS (5 vs 3 days, *p < 0*.*001*). A total of 31 (6.2%) patients were switched from ECLS to LVAD, with significantly more patients in the survivor group [24 (11.7%) vs 7 (2.4%); *p < 0*.*001*]. 43 (8.6%) patients were switched to VV ECMO, but there was no significant difference between survivors and non-survivors. Five (1.0%) patients received heart transplantation and all of them were in the survivor group (*p = 0*.*011*). A total of 307 (61.2%) patients were successfully weaned from ECLS. However, only 206 (41.0%) ultimately survived (*p < 0*.*001*). Causes of death are shown in Table 4.

**Table 4:** Overview of patient outcome: Comparison between survivors and non-survivors.

	All patients (n = 502)	Survival (n = 206)	Non-survival (n = 296)	*p* Value
ECLS duration, days	3 (2–6)	5 (3–7)	3 (1–6)	*<0*.*001*
Complications of ECLS, n (%)	115 (22.9%)	44 (21.4%)	71 (24.0%)	0.52
Leg ischemia Bleeding Cannulation complications	63 (12.6%) 39 (77.7%) 13 (2.6%)	21(10.2%) 19 (9.2%) 4 (2.0%)	42 (14.2%) 20 (6.8%) 9 (3.0%)	0.22 0.31 0.57
Switch to LVAD	31(6.2%)	24 (11.7%)	7 (2.4%)	*<0*.*001*
Switch to VV ECMO	43 (8.6%)	20 (9.7%)	23 (7.8%)	0.52
Heart transplantation	5 (1.0%)	5 (2.4%)	0 (0%)	*0*.*011*
Outcome, n (%)				
Wean-off Survival	307 (61.2%) 206 (41.0%)	206 (100%) 206 (100%)	101 (34.1%) 0 (0%)	*<0*.*001*
Transfusion, units				
RBC Platelets	0 (0–3) 0 (0–0)	0 (0–4) 0 (0–0)	0 (0–3) 0 (0–0)	0.013 0.93
Cause of death, n (%)				
LCO Severe neurologic damage Multiple organ failure Sepsis Bleeding Others	88 (17.5%) 78 (15.5%) 74 (14.7%) 25 (5.0%) 19 (3.8%) 12 (2.4%)			

In the multivariate regression analysis for risk factors associated with mortality, as shown in Table 5, the only independent risk factor was the initial lactate level (Odds ratio 1.01, 95% CI 1.00–1.03, *p = 0*.*032*). No association was seen for SOFA score, LCO state versus inability to wean from ECC, prior cardiac surgery, initial bilirubin level, central cannulation or blood transfusion.

**Table 5:** Multivariate logistic regression analysis for risk factors associated with mortality.

Variable	Odds ratio	95% Confidence interval	*p* Value
SOFA score	1.18	0.98–1.41	0.80
Reason for implantation			
LCO No weaning from ECC	1.66 2.67	0.43–6.48 0.31–22.98	0.47 0.37
Cardiac surgery prior to ECLS	0.94	0.29–3.01	0.92
Initial lactate	1.01	1.00–1.03	*0*.*032*
Initial bilirubin	1.18	0.77–1.81	0.46
Central cannulation	1.38	0.08–24.68	0.83
RBC transfusion	0.97	0.88–1.06	0.46

The boxplots show the correlation between bilirubin levels and dynamic changes of bilirubin levels in survivors and non-survivors (Fig 1) or survivors and weaned patients who did not survive (Fig 2). Bilirubin levels were consistently higher non-survivors and started to rise notably from day 2 in all patients. In survivors bilirubin levels had trended back down on day six after ECLS implantation and stayed at an acceptable level after explantation (p<0.001). However, in the group of weaned patients who did not survive, bilirubin levels remained elevated above baseline or continued to trend upwards during the recorded period (p<0.001). Especially after ECLS explantation, bilirubin levels continued to rise in these patients (p<0.001).

**Fig 3:**
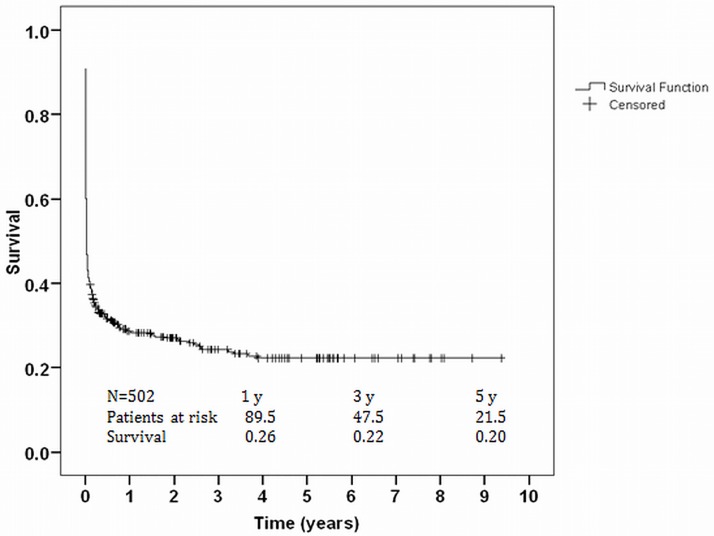
Kaplan–Meier survival curve of patients after ECLS support.

Fig 3 showed the survival curve estimated by Kaplan-Meier method. Survival rate for all patients after ECLS support at 1, 3 and 5 years, including the in-hospital mortalities, were 26%, 22% and 20%, respectively (Fig 3).

## Discussion

The aim of our study was to evaluate the impact of dynamic changes of elevated bilirubin levels over time on survival in a large cohort of 502 patients on ECLS for acute circulatory failure. Notably, our study includes a wide spectrum of patients undergoing ECLS for a variety of reasons. Those included salvage therapy in cardiac arrest, supportive therapy in low cardiac output and extension of therapy if unable to wean off ECC in the operating room.

Elevation in bilirubin generally is a sequela of liver damage. The pathogenesis of liver injury in circulatory failure is attributed to two different etiologies: In chronic congestive heart failure passive congestion results in cardiac cirrhosis (fibrosis) or congestive hepatopathy. In acute cardiogenic shock hypoxic (ischemic) hepatitis, or shock liver, is a result of hypotension or impaired perfusion [13]. Hypoxic hepatitis and congestive hepatopathy have a considerable overlap in laboratory, clinical and histologic features. They are characterized by an increase of aminotransferase levels over 1000 U/L or 50 times the upper limit of normal [14]. Patients with congestive heart failure and preexisting congestive hepatopathy may be predisposed to hypoxic hepatitis by brief episodes of insufficient liver perfusion. One study reported that the manifestations of liver injury, including jaundice and marked increase in prothrombin time, developed after an interval of one to three days, despite the fact that the patients' hemodynamic conditions had returned to baseline [15]. Off note, only half of the patients with hypoxic hepatitis actually had an episode of marked hypotension or shock [14].

In our study population, the etiology of impaired liver function after CPR was hypoxic hepatitis, whereas patients with low cardiac output may have had a combination of hypoxic hepatitis and congestive hepatopathy. Patients with difficulty weaning off ECC in the operating room may have had multifactorial etiology including hypoxic hepatitis and congestive hepatopathy due to preoperative CPR or LCO with possible transient hypoperfusion intraoperatively. Consistent with hypoxic hepatitis we saw rising bilirubin levels starting on day two in our population.

Hypoxic hepatitis and congestive hepatopathy are usually self-limited and treatment is restoring perfusion and cardiac function. Mortality has been reported to be higher in patients who develop hypoxic hepatitis in the intensive care unit who need vasopressor support or who suffer from septic shock, renal failure, or coagulopathy [16], which was evident in most of our patients.

There is huge variability in survival data after cardiac arrest. A recent study in the United States reported an average of 9.6% survival after out-of-hospital arrest and resuscitation [17]. Trends in survival after in-hospital cardiac arrest have reportedly improved over the last decade. A review of the GWTG-Resuscitation registry showed an increased survival to discharge from 13.7% in 2000 to 22.3% in 2009 [18]. Overall survival in our study population was 41%. However, out of 502 patients, only 230 (45.8%) received ECLS during cardiac arrest. Survival in this subgroup was n = 85 (37%). Our results indicate that ECLS can markedly improve survival in cardiac arrest compared to treatment with conventional advanced life support algorithms. Our data are consistent with results of an analysis of the international Extracorporeal Life Support Organization registry between 2003 and 2013, which showed a 42% survival to hospital discharge rate after ECLS [19]. Several other authors have previously tried to identify predictive factors for survival in this specific patient population.

The Sequential Organ Failure Assessment (SOFA) score is a mortality prediction score based on the degree of dysfunction of 6 organ systems and commonly used in intensive care units to track a patient’s status [20]. An initial SOFA score over 11 predicts a mortality of 95%, less than 9 may indicate mortality around 33%. But those data were mainly collected from sepsis trials. Wu et al showed that pre-existing organ dysfunction, quantified by a pre-ECLS SOFA score greater than 14 predicted mortality in their study [21]. In our study, the average pre-ECLS SOFA score was 12, but we did not identify it as an independent risk factor for mortality in multivariate regression analysis.

The Extracorporeal Life Support Organization has recently created the SAVE-Score, a tool to predict survival prior to initiation of ECLS in patients with refractory cardiogenic shock [19]. Their study showed that chronic renal failure, longer duration of ventilation prior to ECLS initiation, pre-ECLS organ failure, pre-ECLS cardiac arrest, congenital heart disease, lower pulse pressure, and lower serum bicarbonate were risk factors associated with mortality. Younger age, lower weight, acute myocarditis, heart transplantation, refractory ventricular tachycardia or fibrillation, higher diastolic blood pressure, and lower peak inspiratory pressure were protective [19]. However, this score has only been validated in 161 patients in an Australian population and larger cohort verification is needed.

Burrell et al. aimed to evaluate factors predicting long-term survival after ECLS support in a small group of 125 patients and identified ischemic heart disease, higher lactate and higher bilirubin levels at initiation of ECLS, and a longer duration of RRT as independent risk factors for decreased survival [22]. Those results partially coincide with our results. We also identified an initially increased lactate level as independent risk factor, but did not see this for bilirubin levels before ECLS implantation. We also found significantly higher initial bilirubin levels upon initiation and on day one in non-survivors.

Several authors have recently evaluated the impact of markers of impaired liver function before initiation of ECLS on survival. However, they only included patients who were unable to wean off ECC after cardiac surgery. A strong inverse association between serum butyrylcholinesterase levels and long- and short-term mortality has been reported by Distelmaier et al in a trial with 191 patients [23]. Roth et al. reported that elevated levels of alkaline phosphatase and bilirubin were sensitive predictors of short- and long-term mortality. However, laboratory tests in their study were routinely performed on admission to the hospital only in patients undergoing cardiac surgery [24].

Neither of these trials assessed markers of impaired liver function dynamically with ongoing ECLS as risk factors for non-survival, nor did they investigate prognostically favorable markers to support weaning off the system. Therefore, we believe our study is the first to evaluate dynamic changes of bilirubin levels as predictors of survival for patients on ECLS. Furthermore, most previously mentioned trials had much smaller patient populations than our study.

Our data indicate that patients with down trending bilirubin levels on ECLS have higher chances of survival and warrant weaning off ECLS support if otherwise hemodynamically stable. In our patient population 15.8% of patients required subsequent hemodynamic support. This included VV ECMO (8.6%), LVAD (6.2%) and heart transplantation (1.0%). There was a statistically significant benefit in survival in patients switched to LVAD or receiving a transplant.

Survivors received significantly longer ECLS. We believe those results are an indication that longer ECLS support allows improved peripheral organ recovery, which may be indicated by downtrending bilirubin levels. Correlation with the SOFA score shows, that normalization of bilirubin levels in otherwise continuously critically ill patients, suggests a mortality reduction of up to 50%.

We feel that initially increased bilirubin levels prior to ECLS are more likely a sign of chronic congestive hepatopathy due to congestive heart failure, rather than being reflective of acute changes due to hypoxic hepatitis. Yang et al found that liver dysfunction is associated with worse survival in patients with advanced heart failure requiring LVAD support. Nevertheless, with improvement of liver function during LVAD support, post-heart transplant survival is similar to patients without previous liver dysfunction [25].

The only independent risk factor for mortality in our multivariate logistic regression analysis was an elevated lactate, but not higher SOFA scores, previous cardiac surgery, central cannulation or blood transfusions. This is most likely explained by the fact that almost half of our study population (45.8%) received ECLS for cardiac arrest and thus where presumed to have previously been in a good state of health.

### Limitations

Our study has several limitations. This was a retrospective study performed at a single medical center, and this design limits the generalizability of these findings. A selection bias as to who was selected for ECLS may have occurred. Bilirubin is one factor among many important variables which may be associated with outcome. Multivariate analysis was applied to control for the effects of other associated variables on survival. Nevertheless, these statistical techniques are limited and will not account for factors not included in the model, which may also affect survival.

## Conclusion

This report suggests that down trending bilirubin levels indicate improved chances of successful weaning and survival in hemodynamically stable patients on veno-arterial ECLS.

Our results might help clinicians’ select appropriate candidates for successful weaning of ECLS. Further randomized studies are needed to evaluate the impact of bilirubin trends on long-term survival.
